# Relationships Between Cardiovascular Autonomic Profile and Work Ability in Patients With Pure Autonomic Failure

**DOI:** 10.3389/fnhum.2021.761501

**Published:** 2021-12-23

**Authors:** Antonio R. Zamunér, Maura Minonzio, Dana Shiffer, Roberto Fornerone, Beatrice Cairo, Alberto Porta, Stefano Rigo, Raffaello Furlan, Franca Barbic

**Affiliations:** ^1^Laboratory of Clinical Research in Kinesiology, Department of Kinesiology, Universidad Católica del Maule, Talca, Chile; ^2^Internal Medicine, IRCCS Humanitas Research Hospital, Milan, Italy; ^3^Department of Biomedical Sciences, Humanitas University, Milan, Italy; ^4^Department of Biomedical Sciences for Health, University of Milan, Milan, Italy; ^5^Department of Cardiothoracic, Vascular Anesthesia and Intensive Care, IRCCS Policlinico San Donato, Milan, Italy

**Keywords:** autonomic nervous system, pure autonomic failure (PAF), orthostatic hypotension, work ability, power spectrum analysis, heart rate variability, blood pressure

## Abstract

Pure autonomic failure (PAF) is a rare disorder belonging to the group of synucleinopathies, characterized by autonomic nervous system degeneration. Severe orthostatic intolerance with recurrent syncope while standing are the two most disabling manifestations. Symptoms may start at middle age, thus affecting people at their working age. The aims of this study were to evaluate the autonomic and work ability impairment of a group of PAF patients and assess the relationships between cardiovascular autonomic control and work ability in these patients. Eleven PAF patients (age 57.3 ± 6.7 years), engaged in work activity, participated in the study. They completed the Composite Autonomic Symptom Score (COMPASS-31, range 0 no symptom-100 maximum symptom intensity) and Work Ability questionnaires (Work Ability Index, WAI, range 7–49; higher values indicate better work ability and lower values indicating unsatisfactory or jeopardized work ability). Electrocardiogram, blood pressure and respiratory activity were continuously recorded for 10 min while supine and during 75° head-up tilt (HUT). Autoregressive spectral analysis of cardiac cycle length approximated as the time distance between two consecutive R-wave peaks (RR) and systolic arterial pressure (SAP) variabilities provided the power in the high frequency (HF, 0.15–0.40 Hz) and low frequency (LF, 0.04–0.15 Hz) bands of RR and SAP variabilities. Cardiac sympatho-vagal interaction was assessed by LF to HF ratio (LF/HF), while the LF power of SAP (LF_SAP_) quantified the vascular sympathetic modulation. Changes in cardiovascular autonomic indexes induced by HUT were calculated as the delta (Δ) between HUT and supine resting positions. Spearman correlation analysis was applied. PAF patients were characterized by a moderate autonomic dysfunction (COMPASS-31 total score 47.08 ± 20.2) and by a reduction of work ability (WAI 26.88 ± 10.72). Direct significant correlations were found between WAI and ΔLF_RR_ (*r* = 0.66, *p* = 0.03) and ΔLF/HF_RR_ (*r* = 0.70, *p* = 0.02). Results indicate that patients who were better able to modulate heart rate, as revealed by a greater cardiac sympathetic increase and/or vagal withdrawal during the orthostatic stimulus, were those who reported higher values of WAI. This finding could be relevant to propose new strategies in the occupational environment to prevent early retirement or to extend the working life of these patients.

## Introduction

Pure autonomic failure (PAF) is a rare neurodegenerative disorder belonging to the group of synucleinopathies (Coon et al., 2019) characterized by orthostatic hypotension with recurrent syncope while standing. The disorder has also been known as Bradbury-Eggleston syndrome, named for the authors of the 1925 seminal description. The prevalence of PAF is about 1--9/1,000,000 in Europe.^1^ Since it is a rare disease, epidemiological information about PAF is limited (Farrell and Shibao, 2020). However, symptoms usually start at middle age or later, thus affecting people that are still at their working age (Kaufmann et al., 2017). Unlike other alpha synucleinopathies, primarily involving central nervous system, the evolution of PAF is slow.

Accumulation of misfolded α-synuclein, also known as Lewy bodies, predominantly in specific peripheral regions of the autonomic ganglia and nerves has shown to be the main pathophysiological mechanism underlying this condition (Hague et al., 1997; Kaufmann et al., 2001; Wang et al., 2020). In addition, noradrenergic cardiac and extra-cardiac denervation, decrease in cardiac parasympathetic modulation, as well as the decrease in the baroreflex sensitivity have been documented in patients with PAF (Furlan et al., 1995; Jain and Goldstein, 2012; Norcliffe-Kaufmann et al., 2018) and may underlie the symptoms of this disorder.

Symptomatic neurogenic orthostatic hypotension with orthostatic syncope is the hallmark of PAF leading patients to seek medical attention. In addition, other signs and symptoms may include, mouth and eye dryness, impaired sweating, mild dysphagia, severe constipation, erectile dysfunction in men, urgency and frequent urination, and thermoregulatory impairment (Mabuchi et al., 2005; Coon et al., 2019). The systemic manifestations of disease, such as anemia and severe supine hypertension, make the clinical pathway of these patients complex and require multidisciplinary treatment approaches (Mabuchi et al., 2005; Barbic et al., 2017; Coon et al., 2019). The exercise capability may be remarkably impaired because of intense asthenia and progressive fatigue. In the severe form of the disease, the drop of blood pressure might also occur also while sitting. Orthostatic hypotension and orthostatic intolerance including presyncope, syncope, brain fog, and cloudy thoughts are also present and may significantly impact on patients’ working activities and quality of life, which ultimately could result in early retirement (Barbic et al., 2014a,^2017^, ^2020^; Coon et al., 2019). However, to the best of our knowledge, no studies have addressed the working performance or the impact of PAF on work ability.

The alteration in neural modulation of heart rate and blood pressure in dysautonomic syndromes may occur before the alteration in mean values of hemodynamic variables (Barbic et al., 2007; Coon et al., 2019; Furlan et al., 2019; Idiaquez et al., 2021), thus suggesting that the assessment of cardiovascular autonomic function might reveal early impairment on mechanisms controlling heart rate and blood pressure. Therefore, elucidating whether the cardiovascular autonomic dysfunction is related to the work ability in PAF patients could be relevant, and might represent a starting point for new strategies to promote occupational environment adjustments to prevent the early retirement of these patients.

Thus, the current study aimed at assessing the autonomic dysfunction and the work ability of a group of still active PAF patients. In addition, we tested the hypothesis that cardiovascular autonomic changes induced by gravitational stimulus (head-up tilt) could be, to some extent, related to the patients’ work ability.

## Materials and Methods

### Participants

Eleven patients with diagnosis of PAF and currently engaged in a paid work were consecutively enrolled in the study. The patients had been referred to the Cardiovascular Autonomic Disorders Unit at Humanitas Research Hospital. Diagnosis of PAF was made according to the current consensus criteria (i.e., presence of neurogenic orthostatic hypotension without a known secondary cause) (Kaufmann, 1996; The Consensus Committee of the American Autonomic Society and the American Academy of Neurology, 1996). Exclusion criteria included the presence of coexisting neurodegenerative diseases, cardiovascular diseases (atrial fibrillation, arrhythmias, and coronary disorders, implanted pacemaker), chronic use of drugs, diabetes mellitus, existing liver, kidney and/or lung disease, and history of alcohol abuse. Participants were also excluded if any motor or cognitive impairment was present.

The study protocol adhered to the principles of the Declaration of Helsinki and was approved by the Local Ethical Review Board (Approval # 1611/2016). The study was funded by the Italian Ministry of Health (Grant RF-2013-02355242). All of the study participants provided their written informed consent.

### Experimental Procedures

All the experiments were conducted in a quiet room with controlled temperature (22–24°C). Participants were asked to void their bladder and rest in the supine position for at least 20 min for stabilization of hemodynamic variables prior to the experiment.

Electrocardiogram (Dual Bio Amp, AD Instruments PtY Ltd., Bella Vista, Australia) and non-invasive blood pressure (Nexfin, SEDA SPA, Trezzano S/Naviglio, Italy) were continuously recorded on a beat-to-beat basis. Respiratory activity was simultaneously recorded by a thoracic belt connected to a pressure transducer (Respiratory Belt, FM, Marazza, Italy). All the signals were digitized at 400 Hz by an analog-to-digital converter, recorded by a data acquisition system (PowerLab 16/35 and LabChart-pro 8.0 software; ADInstruments Pty Ltd., Bella Vista, Australia), and stored on the hard disk of a personal computer for off-line analysis. A catheter was placed on the non-dominant arm for blood withdrawal.

Twenty minutes after adaptation to the experimental setting, baseline data acquisition was initiated, and a first venous blood sample was collected to assess plasma catecholamines (norepinephrine and epinephrine) in the supine position. After 10 min, the participants underwent a 75° head-up tilt (HUT) for 15 min using a motorized tilt table (Low, 2003). Five minutes into the tilt procedure, a second venous blood sample was taken. The cardiovascular autonomic profile of PAF patients was assessed while supine and during HUT, in the absence of pre-syncope symptoms and signs, using synchronous stationary sequences with 256 consecutive measures of RR intervals, systolic arterial pressure (SAP), and respiratory activity. Plasma norepinephrine and epinephrine were determined by high-performance liquid chromatography with electrochemical detection.

### Data Analysis

A detailed description of data processing and analysis has been published elsewhere (Pagani et al., 1986; Malliani et al., 1991; Task Force of the European Society of Cardiology and the North American Society of Pacing and Electrophysiology, 1996; Barbic et al., 2007, 2020). Spectral analysis of RR intervals, SAP, and respiratory activity was performed to obtain the indexes of cardiovascular autonomic profile in both supine and HUT. Synchronous stationary sequences of 300 consecutive beats were found for each time series.

Briefly, two major oscillatory components were identified by the spectrum analysis of the RR intervals: (1) the high-frequency component (HF_RR_ 0.15–0.50 Hz), recognized as an index of the vagal modulation to the sinoatrial node discharge (Pagani et al., 1986; Malliani et al., 1991; Task Force of the European Society of Cardiology and the North American Society of Pacing and Electrophysiology, 1996); and (2) the low-frequency component (LF_RR_, 0.04–0.15 Hz), that when expressed in normalized units (n.u.) is a recognized index of predominantly sympathetic efferent modulation to the sinoatrial node (Pagani et al., 1986; Malliani et al., 1991; Task Force of the European Society of Cardiology and the North American Society of Pacing and Electrophysiology, 1996). The two oscillatory components of RR variability are presented both in absolute units (ms^2^) and normalized units (n.u.). The absolute values of each component correspond to the integral of the oscillatory components. The normalized units of the oscillatory components were obtained by dividing the absolute power of each component by the total variance minus the power of the very-low frequency component (<0.03 Hz) and then multiplying by 100 (Pagani et al., 1986; Malliani, 2000; Furlan et al., 2001). The LF/HF ratio provided a dimensionless index of the instantaneous reciprocal changes of sympatho-vagal modulation of the sinoatrial node discharge (Pagani et al., 1986; Furlan et al., 2000; Barbic et al., 2007, 2014b). The low frequency (LF) oscillatory component of SAP variability (LF_SAP_, ≈0.1 Hz) was computed and is considered a marker of the sympathetic modulation of vasomotor activity (Pagani et al., 1986; Furlan et al., 1990, 2019; Barbic et al., 2007). RR variance (VAR_RR_) and SAP variance (VAR_SAP_) were also calculated to represent overall cardiac and SAP variability, respectively. Spectral analysis of the respiratory signal was performed to assess the main respiratory frequency (Pagani et al., 1986). The cardiac baroreflex sensitivity was estimated via spectral analysis by computing the square root of the ratio of the LF power of RR intervals to the LF power of SAP (α_LF_) (Pagani et al., 1986). A detailed description is provided elsewhere (Pagani et al., 1986; Diedrich et al., 2009; Zamuner et al., 2015).

Besides the values of spectral indices in supine and HUT, changes (Δ) from supine posture to standing were calculated (i.e., values obtained at HUT minus values obtained at supine) for the hemodynamic values and for the indices of cardiovascular autonomic control to account for the adjustment to the orthostatic stimulus.

### Composite Autonomic Symptom Score

The 31-item Composite Autonomic Symptom Score (COMPASS-31) was applied to assess the severity and distribution of symptoms (Sletten et al., 2012). This validated questionnaire provides a markedly abbreviated quantitative measure of autonomic symptoms compared to previously available tools (Suarez et al., 1999). Moreover, the COMPASS-31 is translated and validated in Italian (Pierangeli et al., 2015). Briefly, COMPASS-31 explores six domains of autonomic functions (orthostatic intolerance, vasomotor, secretomotor, gastrointestinal, bladder, and pupillomotor). Besides the domains, a total score comprising the sum of all domains is computed, ranging from 0 (normal) to 100 (the worst condition), with higher scores reflecting greater autonomic dysfunction.

### Work Ability Index

The Work Ability Index (WAI) questionnaire was filled-out by every participant. WAI is a validated tool, widely used in occupation settings, to assess employees’ work ability (Tuomi et al., 1991; de Zwart et al., 2002; El Fassi et al., 2013; Roelen et al., 2014; Rashid et al., 2018; Ilmarinen, 2019) and also identify workers at risk of premature work exit (Roelen et al., 2014). This questionnaire comprises issues related to work demands, worker’s health status and resources. The physician in charge rated the responses according to seven items: (1) current work ability compared to the lifetime best (range 0–10); (2) work ability in relation to the demands of the job (range 2–10); (3) number of current diseases diagnosed by a physician (range 1–7); (4) estimated work impairment due to diseases (range 1–6); (5) sick leave during the past year (range 1–5); (6) own prognosis of work ability 2 years from now (score 1,4,7); and (7) mental resources (range 1–4). Total WAI score ranges from 7 to 49 and allow to classify the employee in four categories based on their ability to work: poor (7–27), moderate (28–36), good (37–43), and excellent (44–49).

### Statistical Analysis

Continuous variables are expressed as mean and standard deviation. The normality of the data was tested via the Shapiro-Wilk test. Paired *t*-test or Wilcoxon tests were used for within group comparison (supine vs. head-up tilt). Spearman correlation analysis was applied to check for the association between spectral indices of RR and SAP time series and WAI. The level of significance was set at 5%. Statistical analyses were conducted using GraphPad Prism™ Software, version 8.0.2 (GraphPad Software, San Diego, CA, United States).

## Results

### Demographic Characteristics, Autonomic Symptoms and Work Ability

Demographic characteristics and job descriptions of the participants are presented in Table 1. PAF patients were middle-aged (57.3 ± 6.7), with normal body mass index. Participants were classified into 3 categories according to their job task and workload as reported in the WAI questionnaire: (1) *blue-collar workers*, defined as those engaged in prevailing manual work activity, usually performed in standing position; (2) *office workers*, defined as employees with professional or semi-professional training whose jobs require a prevailing mental load. They generally work in the sitting position; and (3) *others*, defined as workers characterized by both physical and mental workload, their job tasks may require standing or sitting position.

**TABLE 1:** Demographic characteristics and working profile of the study population.

Variables	PAF patients (*n* = 11)
Age (years)	57.3 ± 6.7
Sex (M/F)	10/1
Weight (kg)	79.2 ± 10.4
Height (cm)	175.7 ± 7.4
BMI (kg/m^2^)	25.7 ± 3.2
**Job task/workload**	
Blue collars/physical, n (%)	3 (27)
Office Workers/mental, n (%)	3 (27)
Other/both physical and mental, n (%)	5 (46)

*BMI, indicates body mass index. Results are expressed as mean ± standard deviation. Blue-collars workers are engaged mostly in manual work activity; Office workers are characterized by prevailing mental load; Others are workers characterized by both physical and mental workload; more details in the text.*

The burden of autonomic symptoms assessed by the COMPASS-31 in PAF patients is shown in Table 2. Data indicated that the PAF patients were characterized by a wide range of autonomic symptoms of moderate-severe intensity.

**TABLE 2:** COMPASS-31 domains and COMPASS-31 total score for the study population.

COMPASS-31 Domains (score range)	Baseline weighted score (*n* = 11)
Orthostatic Intolerance (0–40)	27.3 ± 11.6
Vasomotor function (0–5)	1.2 ± 1.5
Secretomotor function (0–15)	5.3 ± 3.2
Gastrointestinal function (0–25)	8.9 ± 5.6
Bladder function (0–10)	2.4 ± 2.0
Pupillomotor function (0–5)	2.1 ± 1.1
COMPASS-31 total score (0–100)	**47.1 ± 20.2**

*Results are expressed as mean ± standard deviation.*

The WAI domains and WAI total score are presented in Table 3. Moreover, participants’ WAI categories are presented in Table 4. Regarding the classification of work ability, 6 participants (55%) were classified as “poor” work ability, two participants (18%) were classified as “moderate” and three participants (27%) were classified as good. None of the participants were classified as “excellent.” The worst domain (i.e., lowest score) was “Mental Resources,” while the best doma, i.e., highest score) was the “work ability in relation to the job demands.

**TABLE 3:** Work Ability Index (WAI) domains and WAI total score for the study population.

Work ability domains	WAI score (*n* = 11)
Current work ability compared to the lifetime best (0–10)	4.5 ± 2.3
Work ability in relation to the job demands (2–10)	6.1 ± 1.9
Current diseases diagnosed (1–7)	3.7 ± 1.5
Estimated work impairment (1–6)	3.1 ± 1.6
Sick leave in the last year due to the diseases (1–5)	3.3 ± 1.4
Own prognosis of work ability 2 years from now (1,4 e 7)	4.3 ± 2.8
Mental resources (1–4)	2.0 ± 1.1
WAI total score (0–49)	**26.9 ± 10.7**

*Results are expressed as mean ± standard deviation.*

**TABLE 4:** Work ability category according to the total WAI score in pure autonomic failure (PAF) patients.

Work ability index category (score)	PAF patients N (%)
Poor (2–27)	6 (54)
Moderate (28–36)	2 (18)
Good (37–43)	3 (27)
Excellent (44–49)	0 (0)

### Hemodynamic and Cardiovascular Autonomic Profile

Data regarding the hemodynamic and cardiovascular autonomic profile while supine and during orthostatic stimulus are presented in Table 5. As expected, a marked decrease of SAP and diastolic arterial pressure (DAP) values were observed during 75°-HUT. The increase of HR was partially preserved as well as the increase of plasma catecholamines. Despite the significant orthostatic hypotension, all participants completed the 15 min head-up tilt without syncope or pre-syncope. While supine, the variance of RR was low in agreement with the autonomic disease with a prevalence of vagal modulation to the heart.

**TABLE 5:** Hemodynamic variables, spectral indexes of cardiac and vascular autonomic profile while supine and during 75° head-up tilt (HUT).

Variables	Supine	HUT	Supine vs. HUT *p*-value
**Hemodynamic variables**			
HR (beats/min)	67 ± 8	74 ± 9	0.002^a^
SAP (mmHg)	133 ± 15	97 ± 17	<0.0011^a^
DAP (mmHg)	84 ± 16	53 ± 11	0.002^a^
Respiratory rate (rpm)	17 ± 3.3	17 ± 3.4	1.000^a^
**Spectral analysis**			
RR (ms)	913.7 ± 110.7	825.8 ± 103.2	0.001^a^
VAR_RR_ (ms^2^)	395.0 ± 502.9	190.9 ± 286.8	0.060^b^
LF_RR_ (ms^2^)	52.5 ± 91.8	25.4 ± 58.0	0.080^b^
LF_RR_ (n.u.)	25.8 ± 20.6	26.9 ± 24.7	0.270^b^
HF_RR_ (ms^2^)	72.9 ± 87.2	23.1 ± 27.1	0.006^b^
HF_RR_ (n.u.)	68.4 ± 20.2	59.5 ± 25.2	0.230^a^
LF/HF	0.6 ± 0.7	1.0 ± 1.6	0.420^b^
VAR_SAP_ (mmHg^2^)	6.1 ± 5.6	20.8 ± 26.6	0.047^b^
LF_SAP_ (mmHg^2^)	1.0 ± 2.5	2.2 ± 2.8	0.047^b^
α_LF_ (mm/mmHg)	16.0 ± 12.2	3.9 ± 6.7	0.003^b^
Norepinephrine (ng/L)	158.6 ± 65.3	248.3 ± 127.5	0.004^a^
Epinephrine (ng/L)	25.7 ± 30.2	35.3 ± 33.2	0.005^b^

*^a^Within group comparisons using paired t-test. ^b^Within group comparisons using Wilcoxon test. HR, heart rate; SAP, systolic arterial pressure; DAP, diastolic arterial pressure; RR, RR intervals; VAR, variance; LF, low frequency; HF, high frequency; LF, low frequency; HF, high frequency; n.u., normalized units.*

During the gravitational stimulus, the markers of sympathetic modulation to the heart did not increase thus revealing a prevailing impairment of cardiac sympathetic modulation, while the reduction of the cardiac modulation index (HF_RR_) seemed to be still preserved. The index of sympathetic vasomotor control, LF_SAP_, showed a slight increase during the gravitational stimulus suggesting a residual capability to increase the sympathetic modulation to the vessels. The spectral index of baroreflex control (α_LF_) decreased during HUT.

### Relationships Between Work Ability Index and Cardiovascular Autonomic Profile

**FIGURE 1:**
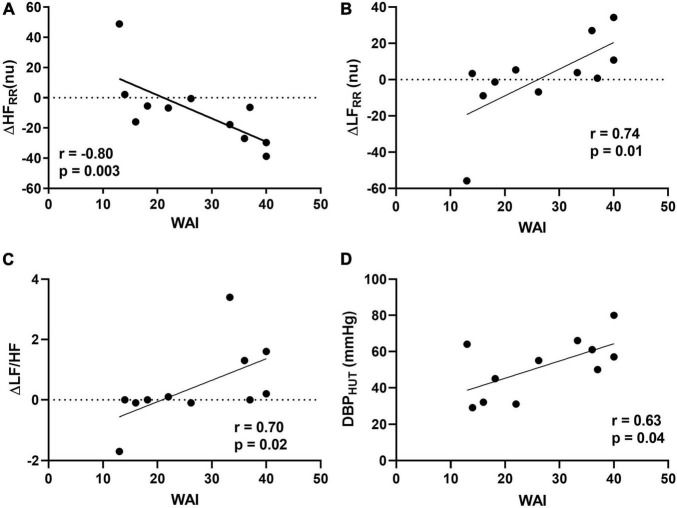
Relationships between Work Ability Index (WAI) and delta changes (Δ) from supine to 75° head-up tilt (HUT) for high frequency (HF) in normalized units **(A)**, low frequency (LF) in normalized units **(B)**, and LF/HF ratio **(C)** and between WAI and diastolic blood pressure (DBP) assessed during HUT **(D)**.

The results of the correlation analysis between WAI and cardiovascular autonomic profile are shown in Figure 1. Significant positive correlations were found between total WAI score and the changes induced by HUT on the spectral indexes of cardiac sympathetic modulation ΔLF_RR_ (*r* = 0.66, *p* = 0.03) and ΔLF_RR_nu (*r* = 0.74, *p* = 0.01). In addition, a significant negative correlation was observed between WAI and the reduction of the spectral index of cardiac vagal modulation ΔHF_*rmRR*_nu (*r* = −0.80, *p* = 0.003). Finally, a significant positive correlation was observed between WAI and the changes induced by HUT of the spectral index of sympatho-vagal modulation to the heart, ΔLF/HF (*r* = 0.70, *p* = 0.02). No significant correlations were observed between WAI and changes in heart rate and blood pressure induced by HUT. A significant positive association was found between WAI and diastolic blood pressure assessed during HUT (*r* = 0.63, *p* = 0.04; Figure 1). Results indicate that the PAF patients with a more preserved adjustment to the orthostatic stimulus, characterized by greater cardiac sympathetic activation and vagal withdrawal were those who reported higher values of WAI.

### Relationships Between Work Ability and Burden of Autonomic Symptoms

**FIGURE 2:**
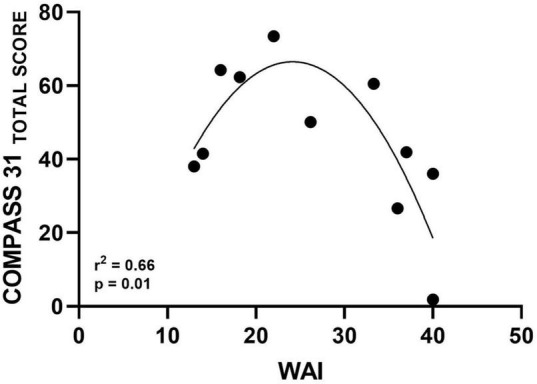
Scatter diagram illustrating significant quadratic relationship between WAI and autonomic symptoms assessed by COMPASS-31 Total Score.

A significant quadratic relationship was found between total WAI and COMPASS-31 total scores (*r*^2^ = 0.66, *p* = 0.01, Figure 2). No correlation was observed between WAI and the single COMPASS-31 domain exploring the orthostatic intolerance of the PAF patients (*r* = −0.47, *p* = 0.15).

## Discussion

The main results of the present study indicated that PAF patients were characterized by a reduced work ability although they were still actively working at the time of the evaluation. In addition, the patients with a more preserved capability to the orthostatic stimulus adjustments, i.e., showing greater cardiac sympathetic activation and greater vagal withdrawal induced by HUT, were those who reported higher values of WAI. To the best of our knowledge, this is the first study addressing the relationships between cardiovascular autonomic control and work ability in patients with PAF.

### Autonomic Burden Symptoms and Work Ability

The values of COMPASS-31 indicated that a medium-severe autonomic impairment characterized the PAF patients of the present study, although they were still working. Indeed, in a previous study by Greco et al. (2017), a COMPASS-31 total score of 16 was suggested as a threshold for early cardiovascular neuropathy.

There are no data on the impact of PAF on work ability, even though the disease often starts often in the middle age thus affecting working-age patients. The orthostatic intolerance with pre-syncope and syncope represents the most common symptoms that induce patients to seek a clinical evaluation (Furlan et al., 1995; The Consensus Committee of the American Autonomic Society and the American Academy of Neurology, 1996; Kaufmann et al., 2017; Coon et al., 2019). However, patients may have difficulties in performing certain job tasks before real problems with orthostatic intolerance were apparent. This may promote early retirement (Barbic et al., 2017) and facilitate disability and depression (Lee and Smith, 2009). In the present study we found that in average, patients with PAF present poor work ability (25.6 ± 10.3), with 55% of the patients being classified as “poor,” 18% as “moderate” and 27% classified still as good.

Several factors may explain the poor work ability observed in these patients. The multiple organ involvement in PAF (Coon et al., 2019) including gastrointestinal and genitourinary dysfunction, supine nocturnal hypertension, nocturia, incontinence, and sexual disorders together with orthostatic hypotension, and recurrent syncope could affect mental and physical performances required by specific jobs. Another possible explanation could be related to abnormal perspiration and thermoregulatory function impairment. The cardiovascular autonomic control, which is markedly impaired in PAF patients, plays a key role in thermoregulatory function (Sawasaki et al., 2001; Castellani and Young, 2016; Greaney et al., 2016). Indeed, abnormal sweating (i.e., hyperhidrosis and anhidrosis) has shown to be present in approximately half of all patients with PAF. This could ultimately increase fatigue and compromise work ability.

Indeed, the reduced work ability reported by PAF patients was similar to what was recently observed in a group of young patients suffering from Postural Orthostatic Tachycardia Syndrome (POTS) (Barbic et al., 2020; Dipaola et al., 2020). Furthermore, the PAF patients in our study reported a significant impairment in mental resources (Table 3), even more pronounced than that observed in patients suffering with POTS (Barbic et al., 2020). This latter is a different dysautonomic syndrome characterized by a severe orthostatic intolerance without orthostatic hypotension, due to an excessive cardiovascular sympathetic modulation (Furlan et al., 1998; Raj, 2013). This observation could be related to the cerebral hypo-perfusion produced by severe orthostatic hypotension (Furlan et al., 1995; Jacob et al., 2018; Coon et al., 2019) that may create dysfunction also during job task performed in the sitting position (i.e., white collars) that characterized some of the PAF patients.

### Relationships Between Work Ability and Cardiovascular Autonomic Control

Results on cardiovascular autonomic control of the present study participants partially corroborate the findings observed by a previous study from our group (Furlan et al., 1995) and the findings of Kaufmann et al. (2017). Both studies (Furlan et al., 1995; Kaufmann et al., 2017) assessed the sympatho-vagal interaction modulating cardiovascular function and the baroreceptor sensitivity in a group of patients with PAF. They found that PAF patients were characterized by diminished values of frequency and time domain indexes of sympathetic and vagal modulation to the heart and sympathetic modulation to the vessels (LF_SAP_), compared to healthy age-matched controls. These non-invasive biomarkers of cardiovascular autonomic control indicated the presence of a complex neural alteration involving both sympathetic and vagal modulation to the heart and sympathetic modulation to the vessels (Diedrich et al., 2003).

The PAF patients of the present study were younger and were characterized by a less severe autonomic pan-dysautonomia compared with patients studied by Furlan et al. (1995). However, the cardiovascular autonomic profile of our patients showed a similar impaired capability to activate the sympathetic modulation to the heart during HUT in the presence of a preserved reduction of cardiac vagal modulation, a slight increase of the sympathetic modulation to the vessels, and a reduction of the spectral index of baroreflex control (α_LF_) (Table 4). The cardiovascular autonomic changes induced by the gravitational stimulus suggest that the neurodegenerative process is incomplete. Indeed, our patients suffering from chronic neurogenic orthostatic hypotension are characterized by an initial high blood pressure drop with a remarkable functional adaptation to low blood pressure during orthostasis. This might be the reason why the PAF patients in our study were still able to perform work activity, although with reduced working performance. Nonetheless, we also found that the lower the diastolic blood pressure during tilt, the lower the WAI. This observation is in keeping with the report by Waldstein et al. (2005), showing that low diastolic blood pressure values were associated with poorer performance on tests of executive function.

It is noteworthy mentioning that the jobs reported by the participants of the present study may require activities in the standing position. Thus, assessing changes of the cardiovascular autonomic control induced by the gravitational stimulus may provide early non-invasive biomarkers of a potential reduction of work ability in PAF patients.

Of interest, the alterations of the markers of cardiovascular autonomic control, as revealed by the gravitational stimulus, seemed to be related to a global working impairment. Indeed, the individual values of the indexes of cardiovascular autonomic control impairment in response to orthostatic stimulus (Head-up tilt) were significantly correlated with the work ability as quantified by the individual values of WAI. These results suggest that an early impairment in cardiovascular autonomic control in response to different environmental stimuli (i.e., the gravitational one) may partially account for the distresses during job tasks and for the reduced perspective of their future working capability.

### Relationships Between Work Ability and Burden of Autonomic Symptoms

A significant quadratic relationship was found in the present study between WAI and burden of autonomic symptoms as assessed by COMPASS-31 total score (Figure 2). This finding shows that participants with better work ability are those with less severe autonomic symptoms. On the other hand, some patients with moderate symptoms also had poor work ability. Although interesting, a clear explanation for this result cannot be provided at present. Several factors may have contributed to this association, such as different work demands among patients with greater and lower symptoms. However, this topic should be further investigated in future studies with a larger sample size. In addition, data indicated the absence of correlation between WAI and the specific orthostatic intolerance domain of the COMPASS-31 questionnaire. It is important to point out that most of our PAF patients, although suffering from moderate-severe orthostatic hypotension, do not complain of a continuous orthostatic intolerance. This could be explained, probably, due to a mechanism of adaptation to the impaired function that might occur due to the slow progression of the disease (Mabuchi et al., 2005). This could also account for the quadratic relationship between autonomic symptoms assessed by COMPASS31 and WAI in our PAF patients.

## Limitation

Some limitations in the present study must be pointed out. First, the sample size is small. This is related to the rarity of this condition with an estimated prevalence in the United States lower than 10,000 people (<0.003%) (Kaufmann et al., 2020). In addition, loss of employment is common in these patients (Barbic et al., 2017) further reducing the number of PAF patients still engaged in a working activity. The presence of only one female in our group represents an additional weakness of the study. Finally, in the present study, we do not include a control group. However, the WAI and COMPASS-31 are well-validated tools to assess work ability and the burden of autonomic symptoms, respectively. In addition, the present study aimed at assessing the relationships between WAI and autonomic impairment as assessed by autonomic symptoms and by the indexes of cardiovascular autonomic control in the same group of patients.

## Conclusion

The PAF patients of the present study were characterized by a moderate-severe autonomic dysfunction and by a global reduction of work ability. Significant correlations were found between WAI and the indexes of cardiovascular autonomic control in response to gravitational stimulus. Results indicated that patients with the preserved capability to modulate heart rate, as revealed by a greater cardiac sympathetic increase and/or vagal withdrawal during the orthostatic stimulus, reported higher WAI values. The non-invasive biomarkers of cardiac autonomic adjustment to the orthostatic stimulus seemed to be related to work ability more than global autonomic symptoms intensity and may help occupational physician in the global management of PAF patients in workplace.

## Data Availability Statement

The raw data supporting the conclusions of this article will be made available by the authors, without undue reservation.

## Ethics Statement

The studies involving human participants were reviewed and approved by the Ethics Research Committee of Humanitas Research Hospital (Approval #1611/2016). The patients/participants provided their written informed consent to participate in this study.

## Author Contributions

RFu and FB conceived and designed research. MM, RFo, and SR performed the experiments. AZ, MM, and BC analyzed the data. AZ, FB, RFu, and AP interpreted results of experiments. AZ and MM prepared the figures. AZ and FB drafted the manuscript. FB, DS, RFu, and AP edited and revised the manuscript. All authors agreed to be accountable for the content of the work.

## Conflict of Interest

The authors declare that the research was conducted in the absence of any commercial or financial relationships that could be construed as a potential conflict of interest.

## Publisher’s Note

All claims expressed in this article are solely those of the authors and do not necessarily represent those of their affiliated organizations, or those of the publisher, the editors and the reviewers. Any product that may be evaluated in this article, or claim that may be made by its manufacturer, is not guaranteed or endorsed by the publisher.

## Footnotes

1
http://www.Orpha.net
